# Thermodynamic and Kinetic Analyses of Iron Response Element (IRE)-mRNA Binding to Iron Regulatory Protein, IRP1

**DOI:** 10.1038/s41598-017-09093-5

**Published:** 2017-08-17

**Authors:** Mateen A. Khan, William E. Walden, Elizabeth C. Theil, Dixie J. Goss

**Affiliations:** 1 Department of Chemistry and Biochemistry, Hunter College of the City University of New York, 695 Park Ave, New York, NY 10065 USA; 2 Department of Life Science, College of Science & General Studies, Alfaisal University, Riyadh, Saudi Arabia; 3 Department of Microbiology and Immunology, University of Illinois at Chicago, Chicago, Illinois 60612-7334 USA; 4 Department of Molecular and Structural Biology, North Carolina State University, Raleigh, NC USA; 5 Children’s Hospital Oakland Research Institute, Oakland, CA 94609 USA

## Abstract

Comparison of kinetic and thermodynamic properties of IRP1 (iron regulatory protein1) binding to FRT (ferritin) and ACO2 (aconitase2) IRE-RNAs, with or without Mn^2+^, revealed differences specific to each IRE-RNA. Conserved among animal mRNAs, IRE-RNA structures are noncoding and bind Fe^2+^ to regulate biosynthesis rates of the encoded, iron homeostatic proteins. IRP1 protein binds IRE-RNA, inhibiting mRNA activity; Fe^2+^ decreases IRE-mRNA/IRP1 binding, increasing encoded protein synthesis. Here, we observed heat, 5 °C to 30 °C, increased IRP1 binding to IRE-RNA 4-fold (FRT IRE-RNA) or 3-fold (ACO2 IRE-RNA), which was enthalpy driven and entropy favorable. Mn^2+^ (50 µM, 25 °C) increased IRE-RNA/IRP1 binding (*K*_d_) 12-fold (FRT IRE-RNA) or 6-fold (ACO2 IRE-RNA); enthalpic contributions decreased ~61% (FRT) or ~32% (ACO2), and entropic contributions increased ~39% (FRT) or ~68% (ACO2). IRE-RNA/IRP1 binding changed activation energies: FRT IRE-RNA 47.0 ± 2.5 kJ/mol, ACO2 IRE-RNA 35.0 ± 2.0 kJ/mol. Mn^2+^ (50 µM) decreased the activation energy of RNA-IRP1 binding for both IRE-RNAs. The observations suggest decreased RNA hydrogen bonding and changed RNA conformation upon IRP1 binding and illustrate how small, conserved, sequence differences among IRE-mRNAs selectively influence thermodynamic and kinetic selectivity of the protein/RNA interactions.

## Introduction

Cellular iron homeostasis is accomplished by the coordinated and balanced expression of iron storage protein ferritin and the iron-uptake protein transferrin receptor. Post-transcriptional regulation of iron homeostasis primarily occurs through the action of iron regulatory proteins, IRP1 and IRP2. IRPs coordinate iron-related gene expression by binding to iron responsive elements (IREs) located in the 5′ or 3′ untranslated regions of mRNAs encoding proteins for iron transport, storage, or utilization; binding blocks either mRNA translation (5′ localized) or degradation (3′ localized)^1–3^. IRPs are ubiquitously expressed, with IRP1 expression dominant in liver, kidney and brown fat, while IRP2 expression is dominant in the central nervous system^4–6^. IRP1 and IRP2 share significant sequence homology^7, 8^ and bind the same family of IRE-mRNAs, but are regulated by iron through different mechanisms. IRP2 also has an additional cysteine rich 73 amino acid domain, the function of this domain is yet unknown^9, 10^. IRP1 is a bifunctional protein, having activity as the aforementioned IRE-RNA binding protein in its apo-form, and as cytosolic aconitase upon assembly of a [4Fe-4S] cluster^3^. Interconversion between these mutually exclusive activities, favoring the aconitase when iron is replete and the apo IRE-RNA binding form in low iron is the basis for gene regulation by IRP1. On the other hand, IRP2 is mono-functional, having activity only as an IRE-RNA binding protein. Iron controls IRP2 activity by regulating IRP2 protein stability. IRPs play an important role in maintaining iron level. Iron homeostasis is closely linked to many diseases such as Alzheimers’s, Parkinson’s, Diabetes, Cancer and Tuberculosis^6, 11, 12^.

Canonical IRE-RNAs are 28–30 nucleotide long stem-loop structures, with a conserved terminal loop sequence, 5′-CAGUGX-3′ (X = U, C, or A), and an unpaired C residue interrupting the stem 5-nucleotides to the 5′ side of the terminal loop sequence. The C and second G residues of the terminal loop sequence form a base pair creating a pseudo-triloop –AGU- in IRE-RNAs^2, 13^. Binding of IRE-RNA with IRP1 occurs mainly through bonds made to the terminal loop and stem interrupting C through two separate binding sites^2^. Approximately two dozen RNA/protein bonds are distributed between the binding sites, giving high affinity and specificity to the interaction. It appears likely that IRP1 binds all naturally occurring IRE-RNA utilizing the same bonding pattern. The structure of the IRE-RNA bound to IRP1 differs from the predominant structure of the RNA in solution, underscoring the importance of conformational flexibility for this high affinity interaction, and raising the possibility that affinity differences observed among these RNA/protein interactions depend in part on the IRE-RNA’s conformational flexibility.

Facile conformation change is also important to IRP1 function, particularly its interconversion between cytosolic aconitase and IRE-RNA binding protein. This interconversion involves large scale domain repositioning and localized conformational changes to form the two RNA binding pockets. Notably, coordination of the iron-sulfur cluster prevents key local protein rearrangements necessary to form the terminal loop binding pocket. Iron regulation of IRP1 function is based on such structural constraints.

Metal ions directly regulate the function of many RNA classes, e.g., tRNA^14, 15^, rRNA^16^, ribozymes^17–21^, riboswitches in bacterial mRNAs, where metals contribute to RNA function and metal sensing^18, 22–25^. Changes in translation of the iron responsive messenger RNAs, dependent on noncoding structures (IRE), are currently ascribed to iron effects on the IRP1 and IRP2, repressor protein, and translation initiation factor (eIF4F), that bind IRE-RNAs and which are modified or degraded by increases in cellular iron concentration^26^. IRE-RNA binds metal ions (Mg^2+^) at specific sites^27^ as do tRNAs, rRNAs, ribozymes and riboswitches. We have previously shown^28, 29^ that metal ions directly affect the IRE-RNA/IRP1 stability and the binding affinity favors ferritin IRE-RNA over aconitase IRE-RNA, illustrating the effects of phylogenetically conserved differences^30^ between the two RNAs. Further, Fe^2+^ (used anaerobically to prevent formation of insoluble hydroxides) could bind to IRE-RNA and act as a ligand to reduce IRP1 binding^28^. This ligand has a further positive effect by increasing binding of eIF4F^31^. Metal ion changes the conformation of ferritin IRE-RNA based on nuclear magnetic resonance^32^ and IRE-RNA containing fluorescence 2-amino purine^31^. Conformational changes of the IRP1 after binding to IRE-RNA has been explain using crystal structure^2, 33^. However, metal ion induced conformational change of IRP1/IRE-RNA complex is not known.

The stability of the IRP1/IRE-mRNA complex is dictated by free energy change that involves both enthalpic and entropic contributions, and thus requires a thermodynamic approach. In this study, we show the effects of temperature on the equilibrium of IRE-RNA binding with IRP1 in the absence and presence of Mn^2+^; Mn^2+^ is an oxygen-stable analogue” for Fe^2+^. *K*_d_ values increased with an increase in temperature. Thermodynamic studies showed a lowering of enthalpy and free energy for the IRE-RNA/IRP1 complex formation in the presence of Mn^2+^, suggesting reduced hydrogen bonding and overall conformational rearrangement during IRE-RNA/IRP1 complex formation. It has been shown from the 3-dimentional crystal structure that hydrogen-bonding and hydrophobic interactions contribute to the stabilization of IRP1/IRE-RNA complexes^2^. Hydrogen bonding contributes to the conformational stability of the molecule; change in the number of hydrogen bonds (Enthalpy change) can cause to conformational changes in the IRE-RNA/IRP1 complex. In order to understand more fully changes in IRE-RNA/IRP1 interactions, we investigated the effect of temperature on the kinetics of IRE-RNA binding with IRP1 in the absence and presence of Mn^2+^. Temperature dependent rate constant values were used to determine the activation energies for FRT and ACO2 IRE-RNA binding to IRP1. Significant decrease in activation energy for binding of FRT and ACO2 IRE-RNA with IRP1 was observed in the presence of Mn^2+^. Decreases in the enthalpic contribution, and lower activation energy for the IRE-RNA/IRP1 complex in the presence of Mn^2+^ provide a path with lower energy barrier.

## Results

### Effect of Temperature on the Interaction of Ferritin and mt-Aconitase IRE-RNA with IRP1

**Figure 1:**
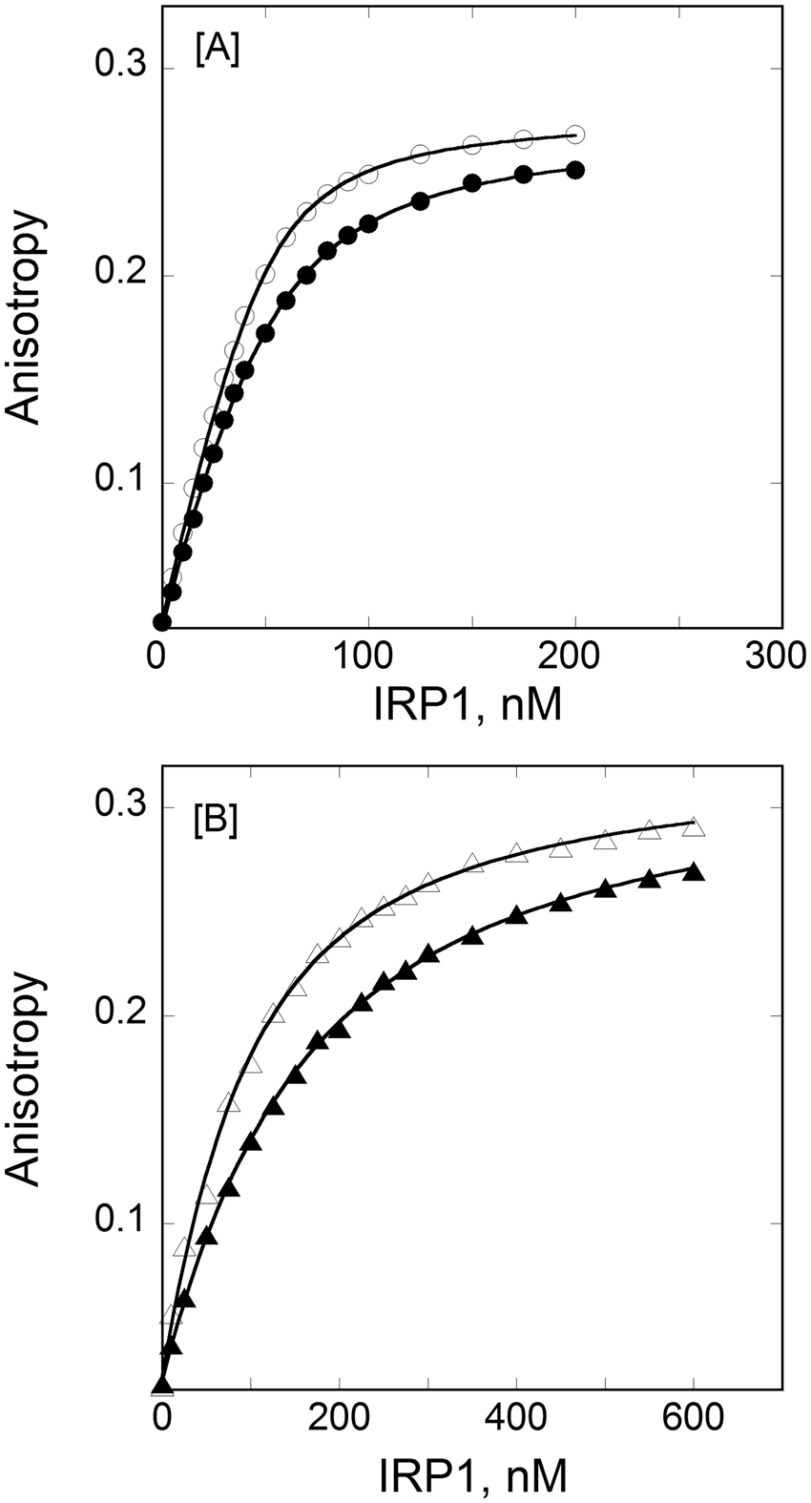
Temperature dependent binding plots for the interaction of IRP1 to IRE-RNA. *Panel A*, temperature affects the binding affinity of FRT IRE-RNA with IRP1 repressor protein. Anisotropy values of FRT IRE-RNA at 10 °C (‒○‒) and 25 °C (–⦁‒) with IRP1 protein are shown. *Panel B*, temperature affects the binding affinity of ACO2 IRE RNA with IRP1 repressor protein. The anisotropy values of ACO2 IRE-RNA at 10 °C (‒∆‒) and 25 °C (‒▲‒) with IRP1 protein are shown. The fluorescein tag IRE-RNA concentration was 50 nM in titration buffer. The excitation and emission wavelength were 490 and 520 nm, respectively. The curves were fit to obtain dissociation constant (*K*_d_) as described in Experimental Procedure. The solid lines are the fitted curves.

**Figure 2:**
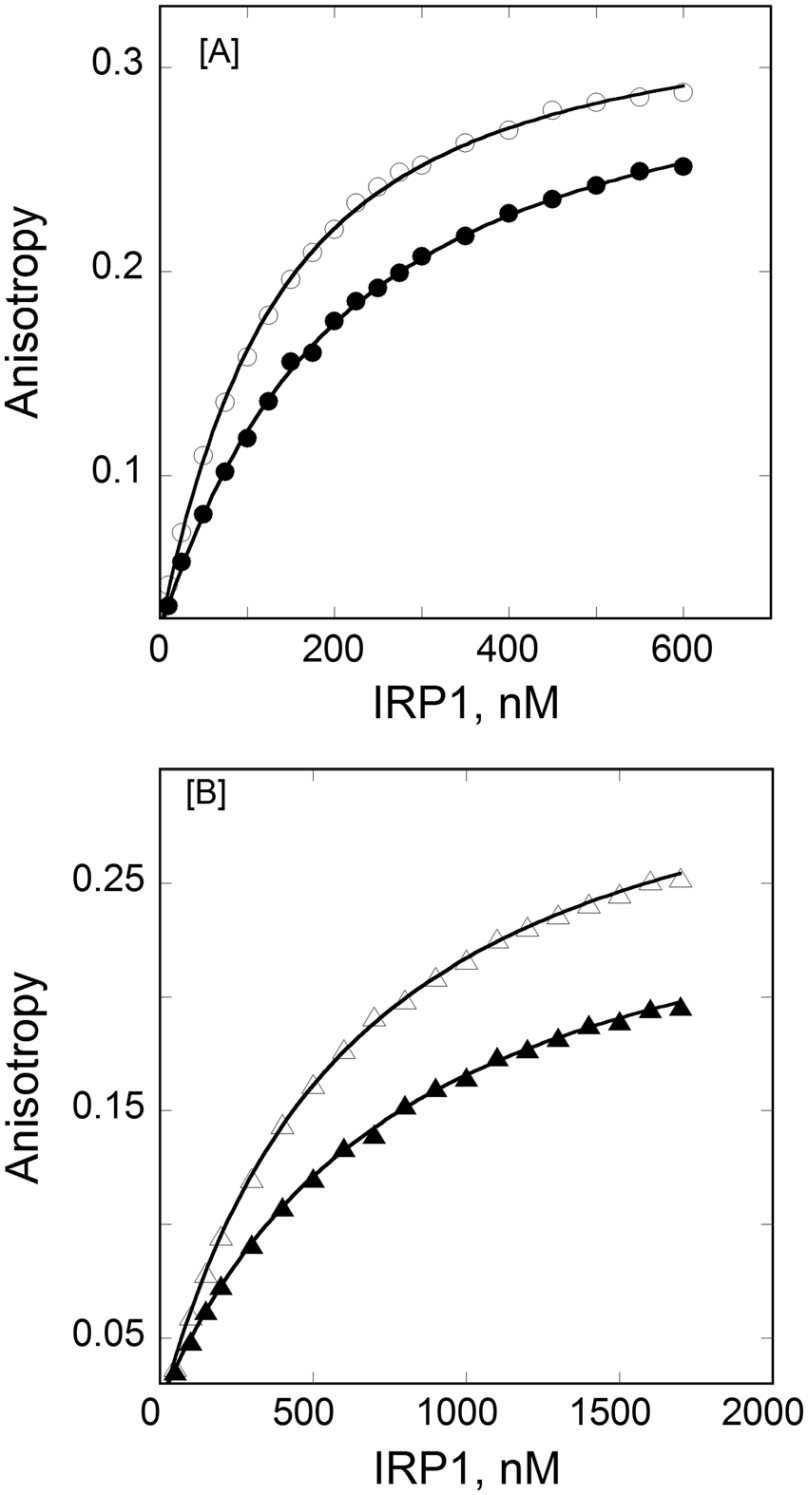
Temperature affects the binding affinity of FRT IRE-RNA with IRP1 repressor protein in presence of metal ions (Mn^2+^). *Panel A*, Anisotropy values of FRT IRE-RNA at 10 °C (‒○‒) and 25 °C (‒⦁‒) with IRP1 protein plus Mn^2+^. *Panel B*, the anisotropy values of ACO2 IRE-RNA at 10 °C (‒∆‒) and 25 °C (‒▲‒) with IRP1 protein plus Mn^2+^. The fluorescein tag IRE-RNA concentration was 50 nM and Mn^2+^ was 50 µM in titration buffer. The excitation and emission wavelength were 490 and 520 nm, respectively. The curves were fit to obtain dissociation constant (*K*_d_) as described in Experimental Procedure. The solid lines are the fitted curves.

Figure 1 shows the representative temperature dependent fluorescence anisotropy measurements for the binding of FRT IRE-RNA and ACO2 IRE-RNA with IRP1. The equilibrium dissociation constants (*K*_d_) for the interactions of the two IRE-RNAs with IRP1 increased with an increase in temperature. *K*_d_ values for the binding of IRP1 to FRT IRE-RNA increased from 4.6 ± 0.2 nM to 19.2 ± 0.4 nM, and for ACO2 IRE-RNA from 55.9 ± 3 nM to 155 ± 4 nM at temperatures ranging from 5 °C to 30 °C (Table 1). Figure 2 shows a representative fluorescence anisotropy plot for the temperature dependent binding of FRT IRE-RNA and ACO2 IRE-RNA with IRP1 in the presence of metal ion (50 µM Mn^2+^). The dissociation constant for the FRT IRE-RNA-Mn^2+^/IRP1 complex increased from 83.3 ± 4 to 193 ± 5 nM while the dissociation constant of ACO2 IRE-RNA-Mn^2+^/IRP1 complex increased from 535 ± 33 nM to 801 ± 36 at temperatures ranging from 5 °C to 30 °C (Table 1). Addition of metal ion decreased the binding affinity of FRT and ACO2 IRE-RNA to IRP1 by 12- and 6-fold, respectively at 25 °C. Dissociation constants for FRT IRE-RNA/IRP1 and FRT IRE-RNA-Mn^2+^/IRP1 complex show a larger effect as compared to ACO2 IRE-RNA/IRP1 and ACO2 IRE-RNA-Mn^2+^/IRP1 complex at the same temperature.

**Table 1:** Temperature dependent equilibrium dissociation constants (*K*_d_) for the interaction of FRT IRE-RNA and ACO2 IRE-RNA with IRP1 protein in the absence and presence of 50 µM Mn^2+^.

Complex	*K*_d_ (nM)					
	5 °C	10 °C	15 °C	20 °C	25 °C	30 °C
FRT IRE-RNA/IRP1	4.6 ± 0.2	7.0 ± 0.2	8.6 ± 0.3	11.7 ± 0.4	14.2 ± 0.3	19.2 ± 0.4
FRT IRE-RNA-Mn^2+^/IRP1	83.3 ± 4	102 ± 6	112.4 ± 5	137.4 ± 6	174 ± 4	193 ± 5
ACO2 IRE-RNA/IRP1	55.9 ± 3	69.6 ± 5	89 ± 3	101 ± 7	129 ± 3.3	155 ± 4
ACO2 IRE-RNA-Mn^2+^/IRP1	535 ± 33	593 ± 29	648 ± 35	701 ± 31	750 ± 32	801 ± 36

### Thermodynamic parameters of Ferritin and mt-Aconitase IRE-RNA complexes with IRP1

**Figure 3:**
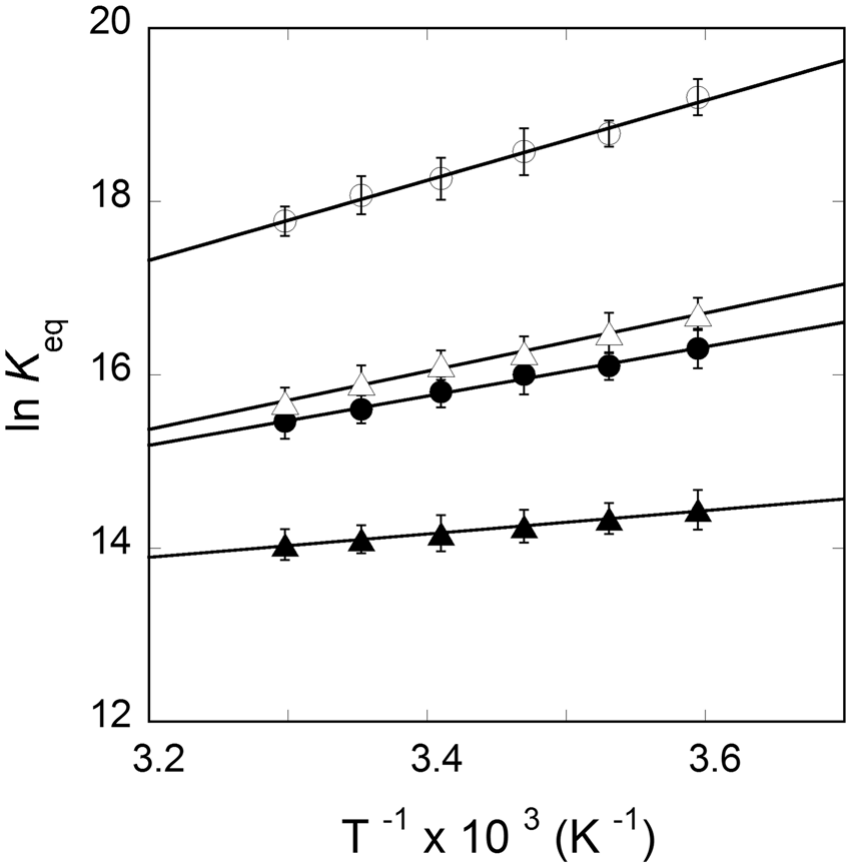
van’t Hoff plots for the interaction of ^FI^IRE-RNA with IRP1 repressor protein in the absence and presence of Mn^2+^. The data are indicated as FRT IRE-RNA (‒○‒), FRT IRE-RNA + 50 µM Mn^2+^ (‒⦁‒), ACO2 IRE-RNA (‒∆‒) and ACO2 IRE-RNA + 50 µM Mn^2+^ (‒▲‒) with IRP1 protein. Enthalpy and Entropy were determined from the slope and intercept of the temperature dependent equilibrium binding measurements, respectively.

The temperature dependence of dissociation constant for the binding of FRT and ACO2 IRE-RNA with IRP1 (Table 1) were used to determine the thermodynamic parameters. Thermodynamic parameters, enthalpy (Δ*H*), entropy (Δ*S*) and free energy (Δ*G*) obtained from van’t Hoff plots of - ln *K*_eq_ *versus* reciprocal of temperature (T^−1^) are shown in Fig. 3 and Table 2. Thermodynamic data showed that binding of FRT and ACO2 IRE-RNA with IRP1 was enthalpy driven and entropy favorable. Addition of metal ion (Mn^2+^) significantly changed the enthalpic and entropic contributions for the binding of FRT IRE-RNA and ACO2 IRE-RNA with IRP1. The ∆*H* and ∆*S* values for the binding of the FRT IRE-RNA with IRP1 were −38.4 ± 2.4 kJ/mol and 21.1 ± 1.3 J/mol/K, respectively, whereas for binding of FRT IRE-RNA to IRP1 in presence of Mn^2+^ the values were −23.6 ± 1.3 kJ/mol and 51.0 ± 3.3 J/mol/K, respectively. The thermodynamic analyses in the absence of Mn^2+^ showed that the binding of FRT IRE-RNA to IRP1 is 88% enthalpically driven and 15% entropy favorable with a 73% greater enthalpic contribution to ∆*G* at 25 °C. Similarly, the binding of ACO2 IRE-RNA with IRP1, showed 71% enthalpically and 29% entropically favorable with a 42% greater enthalpic contribution to ∆*G* at 25 °C. Addition of Mn^2+^ decreases the enthalpic contribution to about 61% and increases the entropic contribution to about 39% for FRT IRE-RNA binding to IRP1. Further, Mn^2+^ decreases the enthalpic contribution to about 32% and increases the entropic contribution to about 68% for ACO2 IRE-RNA binding to IRP1. These data suggest that metal ion induces a conformational change in FRT and ACO2 IRE-RNA/IRP1 complexes resulting in reduced hydrogen bonding. The ∆*G* values at 25 °C were calculated from Eq. 3. The free energy value for the binding of FRT IRE-RNA is higher than ACO2 IRE-RNA with IRP1. Addition of Mn^2+^ further decreased in Δ*G* value for FRT IRE-RNA and ACO2 IRE-RNA binding to IRP1 (Table 2).

**Table 2:** Thermodynamic parameters of enthalpy (Δ*H*), entropy (Δ*S*) and free energy change (Δ*G*) for the interactions of FRT and ACO2 IRE-RNA with IRP1 protein in the absence and presence of 50 µM Mn^2+^.

Complex	Δ*H* (kJ/mol)	Δ*S* (J/mol/K)	Δ*G* (kJ/mol)
FRT IRE-RNA/IRP1	−38.4 ± 2.4	21.1 ± 1.3	−44.0 ± 2.8
FRT IRE-RNA-Mn^2+^/IRP1	−23.6 ± 1.3	51.0 ± 3.3	−38.0 ± 2.1
ACO2 IRE-RNA/IRP1	−27.9 ± 1.4	38.5 ± 2.7	−39.3 ± 1.9
ACO2 IRE-RNA-Mn^2+^/IRP1	−11.2 ± 0.6	79.7 ± 4.8	−34.0 ± 2.2

### Effect of Temperature on the Kinetics of Ferritin and mt-Aconitase IRE-RNA Binding to IRP1

**Figure 4:**
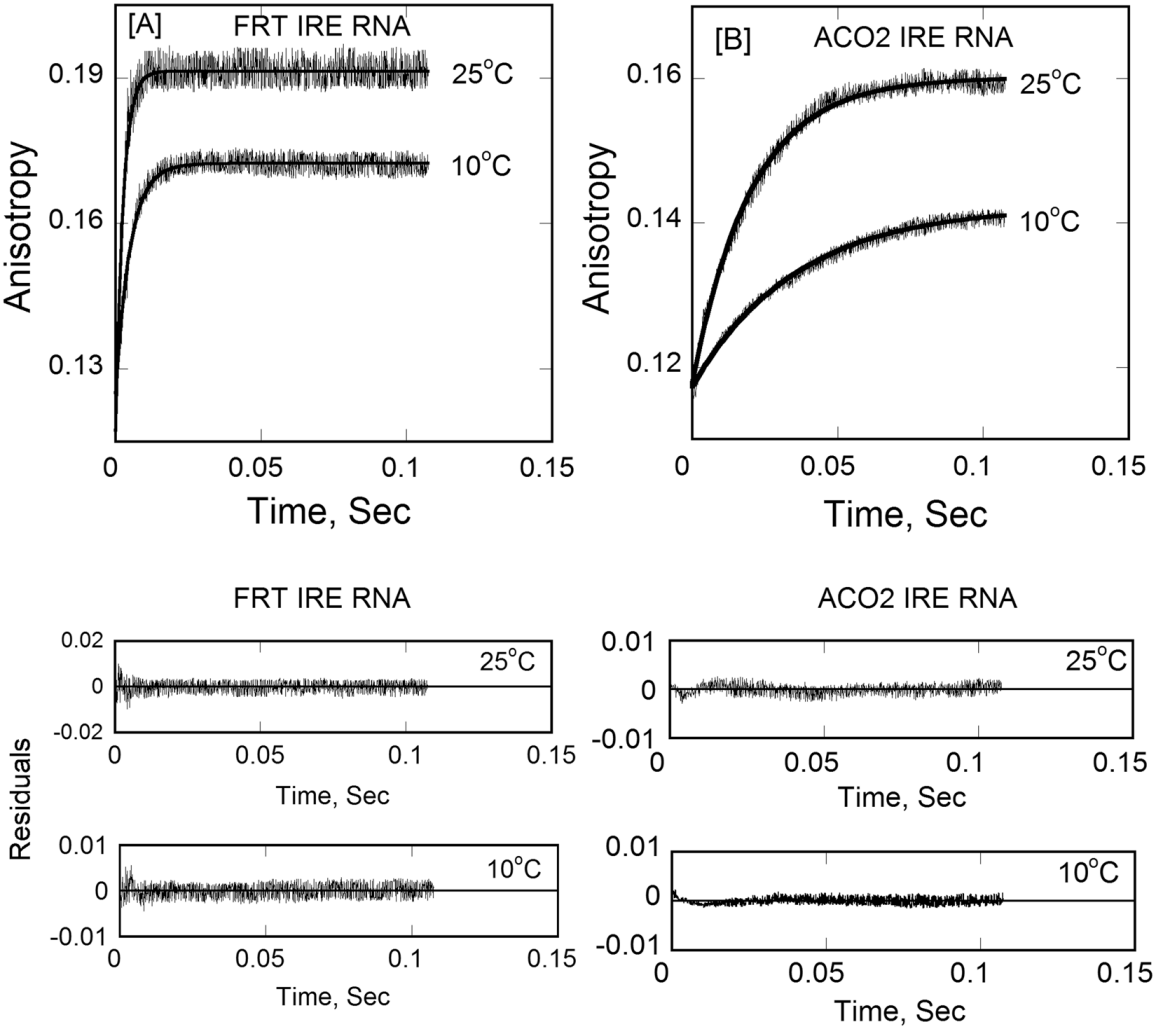
Stopped-flow kinetic measurements of binding of ^FI^FRT IRE-RNA and ^FI^ACO2 IRE-RNA with IRP1 protein. Representative kinetic data show the time-dependent increase in anisotropy after (**A**) FRT IRE-RNA and (**B**) ACO2 IRE-RNA had been mixed with IRP1 at temperature 25 °C and 10 °C. FRT and ACO2 IRE-RNA concentrations was 50 nM (final) and the IRP1 concentration was 1 µM (final). The solid line represents the fitted curve drawn through the data points was fit by assuming a single-exponential process. Residuals for the exponential fits are shown in the bottom panels. The experimental conditions are described in Experimental Procedures.

**Figure 5:**
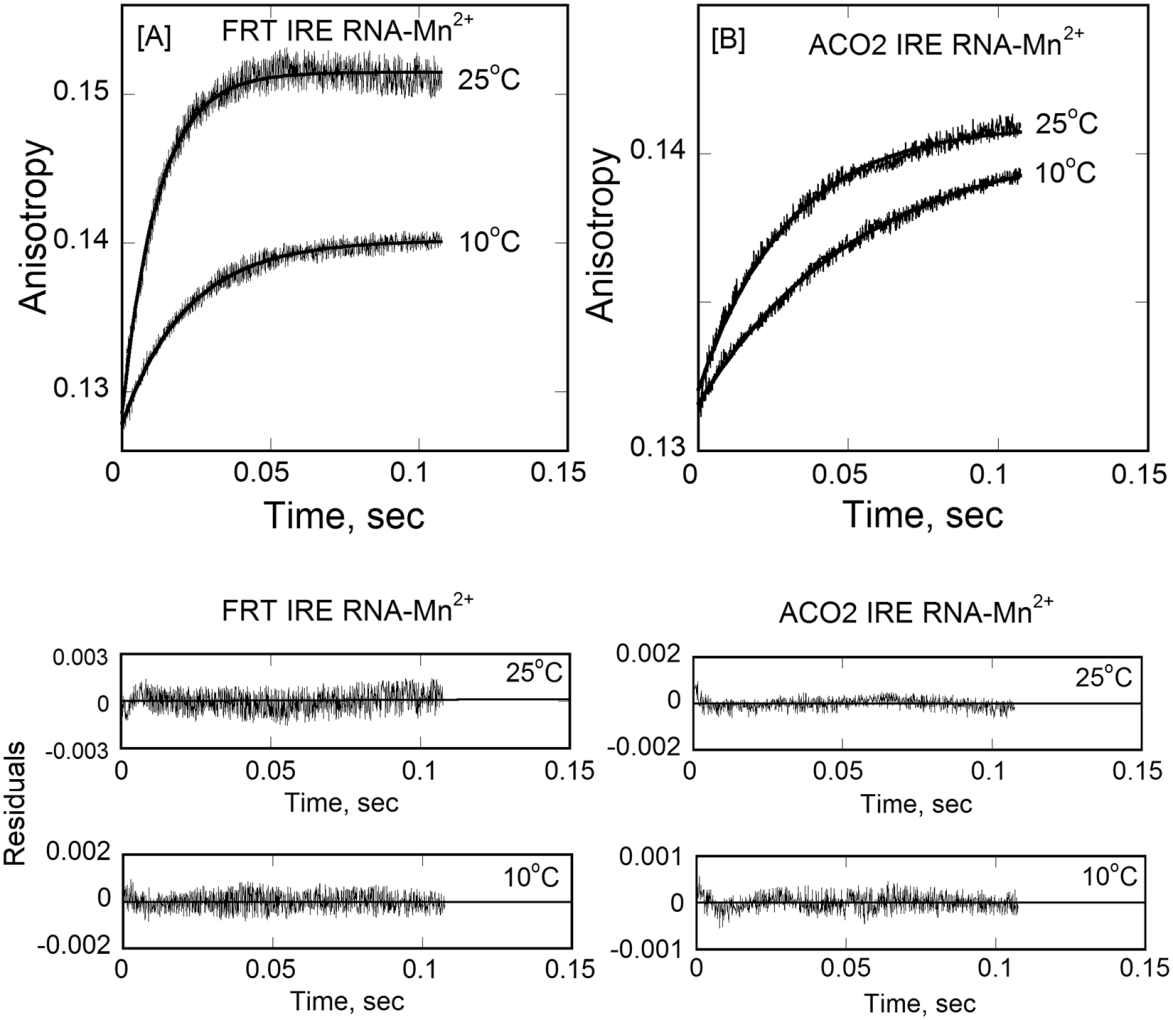
Temperature dependent kinetics of IRP1 binding with ^FI^FRT IRE-RNA-Mn^2+^ and ^FI^ACO2 IRE-RNA-Mn^2+^. Representative kinetic data show the time-dependent increase in anisotropy after (**A**) FRT IRE-RNA-Mn^2+^ and (**B**) ACO2 IRE-RNA-Mn^2+^ had been mixed with IRP1 protein at temperature 25 °C and 10 °C. Concentration of Mn^2+^ was 50 µM. FRT and ACO2 IRE-RNA concentrations was 50 nM (final) and the IRP1 concentration was 1 µM (final). The solid line represents the fitted curve for single-exponential function. Residuals for the fits are shown in the bottom panels. The experimental conditions are described in Experimental Procedures.

**Figure 6:**
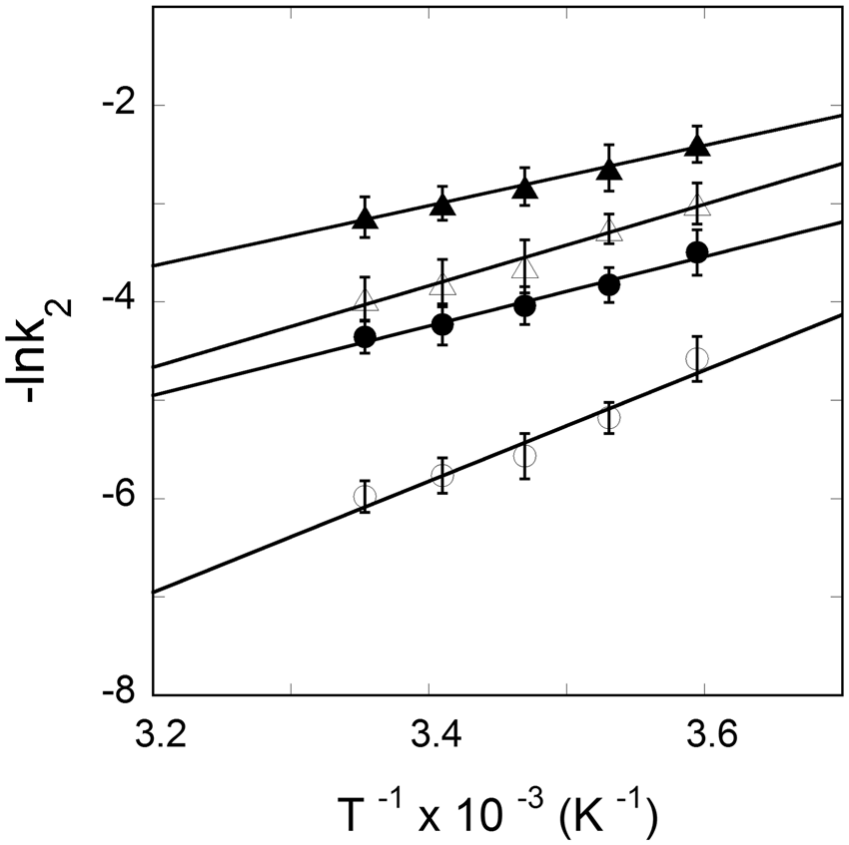
Determination of activation energies for the interaction of IRP1 with FRT and ACO2 IRE-RNA in the absence and presence of Mn^2+^. Rate constant values for FRT IRE-RNA (‒○‒), FRT IRE-RNA-Mn^2+^ (‒⦁‒), ACO2 IRE-RNA (‒∆‒) and ACO2 IRE-RNA-Mn^2+^ (‒▲‒) with IRP1 protein at different temperatures were used to construct an Arrhenius plot according to Eq. 5. Concentration of IRE-RNA, IRP1 and Mn^2+^ were 50 nM, 1 µM and 50 µM, respectively. The activation energy was calculated from the slope of the fitted linear plot of ln *k versus* 1/T (kelvin). Data points in the plot of ln *k versus* 1/T were obtained from three independent experiments and the average value of the experimental data is reported.

We have previously^29^ determined the kinetic rates for the binding of FRT and ACO2 IRE-RNA with IRP1 in the absence and presence of metal ion. Here, we further examine the effect of temperature on the kinetic rate of FRT and ACO2 IRE-RNA binding with IRP1 in the absence and presence of Mn^2+^ by stopped-flow (rapid-mixing) experiments; they were conducted using high concentration of IRP1 and limiting concentrations of FRT IRE RNA and ACO2 IRE RNA to ensure that the bimolecular combination of IRP1 and FRT and ACO2 IRE RNA was pseudo-first order. Stopped-flow data for a typical reaction of IRP1 binding to FRT or ACO2 IRE-RNA were plotted as the anisotropy change *versus* time are shown in Fig. 4. The kinetic data were fitted to a single-exponential equation using nonlinear regression analysis as described in Experimental Procedures. Reaction were consistent with single-exponential fitting over the entire time course of the measurements, as described previously^29^. Analyses of the data using a double exponential components did not improve the fitting results (not shown). The values of the observed rate constants for complexes of IRP1 with FRT or ACO2 IRE-RNA increased with an increase in temperature (Table 3). The residuals representing the deviation between the calculated and experimental data (Fig. 4, bottom panel) indicate that the single-exponential function fits the points over the entire time course of measurements. The kinetic data showed that FRT IRE-RNA/IRP1 binding rate (*k*_2_ = 397 ± 8.5 s^−1^) and ACO2 IRE-RNA/IRP1 binding rate (*k*_2_ = 53 ± 2.4 s^−1^) at 25 °C were ~4.0- and 2.5-fold faster than at 5 °C (FRT IRE-RNA/IRP1, *k*_2_ = 98 ± 4.4 s^−1^ and ACO2 IRE-RNA/IRP1, *k*_2_ = 20 ± 1.8 s^−1^; Table 3). The stopped-flow kinetic data revealed that at all five temperatures FRT IRE-RNA had the higher rate constants compared to the ACO2 IRE-RNA binding to IRP1. FRT IRE-RNA/IRP1 binding had a rate constant 4–8-fold higher than that of ACO2 IRE-RNA/IRP1 (Fig. 4). Figure 5 shows the temperature dependent change in kinetic rates for the binding of FRT and ACO2 IRE-RNA with IRP1 in the presence of Mn^2+^. In order to determine the activation energy (*E*_a_) of the FRT and ACO2 IRE-RNA binding to IRP1 in the absence and presence of Mn^2+^, the temperature dependent rate constant (Table 3) values were used to construct Arrhenius plots (Fig. 6) according to Eq. 5. The activation energies were obtained from the slope of the linear fit of the plot of ln *k versus* 1/T. The activation energies for IRP1 binding to FRT and ACO2 IRE-RNA were 47 ± 2.5 and 35 ± 2.0 kJ/mol, respectively. Addition of Mn^2+^ lowers the activation energies for the FRT IRE-RNA/IRP1 and ACO2 IRE-RNA/IRP1 complexes to 29.0 ± 1.6 and 25 ± 1.2 kJ/mol, respectively (Table 3). The overall lower activation energy for FRT IRE-RNA/IRP1 and ACO2 IRE-RNA/IRP1 complexes in the presence of Mn^2+^ binding suggests that metal ion succeeds in conformational alteration of the IRE-RNA/IRP complex, causing release of IRP1 repressor protein and allows translation initiation factor binding and an increase in protein biosynthesis^31^.

**Table 3:** Temperature dependent kinetic rate constants for the interaction of FRT and ACO2 IRE-RNA with IRP1 in the absence and presence of 50 µM Mn^2+^.

Complex	Observed Rate Constant, *k*_2_ (s^−1^)					*E*a (kJ/mol)
	5 °C	10 °C	15 °C	20 °C	25 °C	
FRT IRE-RNA/IRP1	98 ± 4.4	178 ± 5.7	263 ± 6.5	319 ± 7.6	397 ± 8.5	47.0 ± 2.5
FRT IRE-RNA-Mn^2+^/IRP1	33 ± 2.1	46 ± 2.8	57 ± 3.0	69 ± 2.9	78 ± 3.6	29.0 ± 1.6
ACO2 IRE-RNA/IRP1	20 ± 1.8	26 ± 1.6	38 ± 2.0	45 ± 2.1	53 ± 2.4	35.0 ± 2.0
ACO2 IRE-RNA-Mn^2+^/IRP1	11 ± 0.7	14 ± 0.6	17 ± 1.4	20 ± 0.9	23 ± 1.7	25.0 ± 1.2

## Discussion

In this study, we have examined the detailed temperature dependent equilibria and kinetics for complex formation of ferritin and mitochondrial aconitase IRE-RNAs binding to IRP1. Previously^28^ we had shown that the IRP1 complex with ferritin and mitochondrial aconitase IRE-RNAs is destabilized by metal ions, particularly Fe^2+^ and Mn^2+^. Using fluorescence anisotropy measurements, we have shown that equilibrium dissociation constant increased with an increase in temperature in absence and presence of metal ions. Thermodynamic parameters showed that FRT and ACO2 IRE-RNA binding to IRP1 is enthalpy driven with a large negative ∆*H* and a small positive ∆*S*, whereas addition of Mn^2+^ decreased the enthalpic contribution and increased the entropic contribution for both FRT and ACO2 IRE-RNA binding to IRP1. The decrease in enthalpy and increase in entropy contribution for the two IRE-RNA/IRP1 complexes in the presence of metal ion suggest reduced hydrogen bonding. Binding of FRT and ACO2 IRE-RNA to the IRP1 is characterized by both favorable enthalpy and favorable entropy in the temperature range studied (Table 2). An enthalpy decrease resulting from a comparison of the binding enthalpies for FRT/IRP1 and FRT-Mn^2+^/IRP1, equals Δ*H*_FRT/IRP1_ − Δ*H*_FRT-Mn_^2+^_/IRP1_ = −14.8 kJ/mol, and for ACO2/IRP1 and ACO2-Mn^2+^/IRP1 equal Δ*H*_ACO2/IRP1_ − Δ*H*_ACO2-Mn_^2+^_/IRP1_ = −16.7 kJ/mol. These enthalpy differences are comparable for the enthalpy change per hydrogen bond, Δ*H*_B_ ~ −13 kJ/mol, which is a typical value for hydrogen bonds^34^. This observation can serve as an indicator of decrease in hydrogen bonding for the IRE-RNA-IRP1 interactions with decrease in enthaply. Our enthalpy data is further supported by the free energy data for the contribution of hydrogen bonding between IRE-RNA and IRP1 protein. Addition of metal ion decreased binding free energy, Δ*G*, of about −6.0 kJ/mol and the binding affinity for FRT IRE-RNA/IRP1 complex by 12-fold. Similarly, Mn^2+^ decreased free energy, Δ*G*, of about −5.0 kJ/mol and the binding affinity by 6-fold for ACO2 IRE-RNA/IRP1. This difference corresponds to the free energy difference of about 5–6 kJ/mol, which is a typical value of a single hydrogen bond or a salt bridge formed between a protein and RNA^35, 36^. This force (hydrogen bonding) contributes to the conformational stability of the molecule. IRP1 contains two binding pockets that make 22 bonds with IRE-RNA. The conversion of IRP1 from an aconitase to an IRE-RNA/IRP1 complex requires extensive changes in protein and RNA conformation^2^. The conformational change of the IRE-RNA/IRP1 complex may also be controlled thermodynamically as reported previously for RNA-protein interactions for U1A protein binding to stem/loop II of U1 snRNA^37, 38^. This conformational change most likely also plays a role in kinetics of the FRT and ACO2 IRE-RNA with IRP1 complexes.

The mechanism of the iron effect in cells is unknown; when cells or animals are treated with iron, usually as iron salts or heme, IRE-mRNAs are shifted to polysomes (e.g. ferritin) or degraded (e.g. transferrin receptor) along with degradation of RNA-free repressor proteins, and increased insertion of [4Fe-4S] clusters into the RNA- free form of one of the protein repressors (IRP1), which becomes cytoplasmic aconitase^26, 39^. In order to respond rapidly to changes in the cellular Fe^2+^ concentrations, repression and de-repression of IRE-mRNA must occur on a relatively rapid time scale and modulation of these rates can have important consequences for iron homeostasis. The kinetic rates for the interaction of FRT and ACO2 IRE-RNA with IRP1 follow a single, bimolecular binding step mechanism such as a fast binding followed by a conformational change. Kinetic mechanism is consistent with our previously determined rates of reaction for FRT IRE-RNA and ACO2 IRE-RNA with IRP1^29^. Using stopped-flow kinetics, we have determined the kinetic rate at different temperature in the absence and presence of metal ion for the binding of FRT and ACO2 IRE-RNA with IRP1, which has not previously been measured. Metal ion affected both the association rate and the dissociation constant for IRP1 interaction with FRT and ACO2 IRE-RNA. These results suggest the interactions with metal ions affect the intermediate conformational change in complex formation and reflect a direct sensing of the ion by IRE-RNA, analogous to a riboswitch. Metal ion destabilization of messenger IRE-RNA/protein repressor complexes competes with the stabilization conferred by the very large number of bonds between the protein and the IRE-RNA and the stability of the RNA fold. Selective, metal induced destabilization of IRE-RNA/repressor protein complexes as well as other selective, metal-RNA interactions^22–25, 28^, emphasize the sensitivity of RNA structure/function to the environment.

## Methods

### Preparation of Protein and RNA

The recombinant rabbit IRP1 protein was expressed in yeast and isolated using methods as described previously^29, 40^. The concentration of protein was determined by a Bradford assay with bovine serum albumin as standard^41^ using a Bio-Rad protein assay reagent (Bio-Rad Laboratories, CA). RNA oligonucleotides labeled with fluorescein at the 5′ terminus (^FI^IRE-RNA) were synthesized by Gene Link, Inc. Hawthorne, New York, U.S.A. The ferritin IRE-RNA (nucleotide, ^FI^-GUUCUUGCUUCAACAGUGUUUGAACGGAAC) and mitochondrial aconitase IRE-RNA (nucleotide, ^FI^-CCUCAUCUUUGUCAGUGCACAAAAUG GCG) were used for equilibrium and kinetic studies as described previously^29^. FRT IRE-RNA or ACO2 IRE-RNA was dissolve in buffer containing 40 mM HEPES/KOH, pH 7.2 and RNA was melted and reannealed by heating to 85 °C for 15 min with slow cooling to 25 °C^28, 42^. The concentrations of FRT IRE-RNA and ACO2 IRE-RNA were determined spectrophotometrically using the absorbance at 260 of 40 µg/ml RNA as 1. The purity of synthesized RNAs were checked by measuring the absorbance ratio, A_260/280 nm_, and the absorbance ratio was 2.1.

### Fluorescence Anisotropy Measurements

Temperature dependent fluorescence anisotropy experiments for the binding of FRT and ACO2 IRE-RNA with IRP1 protein were performed using an L-format detection configuration of a Spex Fluorolog τ2 Spectrofluorimeter equipped with excitation and emission polarizers. The anisotropy values for each sample were monitored at an excitation wavelength of 490 nm and emission wavelength of 520 nm. The excitation and emission slits were 4 and 5 nm, respectively. The excitation slits were chosen to avoid photo-bleaching, and the absorbance of the sample at the excitation wavelength was less than 0.02 to minimize the inner-filter effect. Emission spectra were corrected for the wavelength dependent lamp intensity and monochromator sensitivities. In order to study the temperature dependence of the FRT IRE-RNA/IRP1 and ACO2 IRE-RNA/IRP1 complexes, samples were thermostatically adjusted at different temperatures (5, 10, 15, 20, 25 and 30 °C), using a 10-mm path length quartz cuvette. 100 nM of 5′ fluorescein labeled FRT IRE-RNA or ACO2 IRE-RNA was incubated with varying concentrations of IRP1 (0.0–1 mM) in the absence and presence of 50 µM Mn^2+^ (an O_2_-resistant surrogate) in the titration buffer, 40 mM HEPES/H+ , pH 7.2, 100 mM KCl. We use Mn^2+^ as an oxygen-resistant surrogate for Fe^2+^; in addition, Mn^2+^ has effects similar to Fe^2+^ for the IRP1/IRE-RNA complex formation^28^, to facilitate experiments in the presence of air^43^. All samples were pre-incubated for at least 15 minutes prior to the titration experiments. The FRT IRE-RNA/IRP1 and ACO2 IRE-RNA/IRP1 interaction experiments were performed at temperatures, 5, 10, 15, 20, 25 and 30 °C; sample temperatures were measured using a thermocouple device inside the cuvette. Interactions of FRT IRE-RNA/IRP1 and ACO2 IRE-RNA/IRP1 complexes were measured by the enhancement in ^FI^IRE-RNA anisotropy. The anisotropy data were fitted to Equation (1) to determine the dissociation equilibrium constant^44–46^.

1 $${{\rm{r}}}_{{\rm{obs}}}={{\rm{r}}}_{{\rm{\min }}}+\{({{\rm{r}}}_{{\rm{\max }}}-{{\rm{r}}}_{{\rm{\min }}})/({\rm{2x}}[{}^{{\rm{FI}}}{\rm{I}}{\rm{RE}} \mbox{-} {\rm{RNA}}])\}{\{b}^{2}-4[{}^{{\rm{FI}}}{\rm{I}}{\rm{RE}} \mbox{-} {\rm{RNA}}]{[{\rm{IRP1}}]}^{0.5}\}$$

${\rm{b}}={K}_{d}+[{}^{{\rm{FI}}}{\rm{I}}{\rm{RE}} \mbox{-} {\rm{RNA}}]+[{\rm{IRP}}1]$, where r_obs_ is the observed anisotropy at any point in the titration curve, r_min_ is the minimum observed anisotropy in the absence of IRP1 protein, and r_max_ is the maximum anisotropy at saturation; [^FI^IRE-RNA] and [IRP1] are the concentrations of these components. *K*_d_ is the equilibrium dissociation constant. KaleidaGraph software (version 2.1.3; Abelbeck Software) was used to obtain the *K*_d_ values and standard errors for parameters using nonlinear least square fitting of the data. For all equilibrium measurements, three independent titration experiments were performed, and the value reported is the average.

### Thermodynamic Analyses of FRT IRE-RNA and ACO2 IRE-RNA Binding to IRP1

In order to study the temperature dependence of FRT IRE-RNA and ACO2 IRE-RNA interaction with IRP1 in the absence and presence of 50 µM Mn^2+^, the samples were thermostatically adjusted at different temperatures, 5, 10, 15, 20, 25 and 30 °C, respectively. The temperature dependent *K*_eq_ was used to determine enthalpy (Δ*H*), entropy (Δ*S*), and free energy (Δ*G*) of FRT and ACO2 IRE-RNA binding to IRP1 in the absence and presence of Mn^2+^. The thermodynamic parameters of FRT IRE-RNA and ACO2 IRE-RNA binding to IRP1 were analyzed according to the van’t Hoff equation.

2 $$-R{\rm{T}}\,\mathrm{ln}\,{K}_{{\rm{eq}}}={\rm{\Delta }}H-{\rm{T}}{\rm{\Delta }}S$$

3 $${\rm{\Delta }}G=-R{\rm{T}}\,\mathrm{ln}\,{K}_{{\rm{eq}}}$$

where *R* and T are the universal gas constant and absolute temperature, respectively. *K*_eq_, the equilibrium constant, was determined as a function of temperature. Δ*H* and Δ*S* were obtained from the slope and intercept of the plot of ln*K*_eq_ *versus* 1/T (kelvin). Δ*G* was determined at 25 °C using Equation 3.

### Stopped-Flow Anisotropy Measurements

Temperature dependent kinetic measurements of FRT IRE-RNA and ACO2 IRE-RNA interactions with IRP1 protein at temperatures, 5, 10, 15, 20 and 25 °C were performed on an OLIS RSM 1000 stopped-flow spectrometer with polarizers. The dead time of the instruments was 1-ms. Excitation wavelength was 490 nm and emission wavelength was 520 nm for fluorescein labeled FRT IRE-RNA and ACO2 IRE-RNA. In each experiment, 1000 pairs of data points were collected throughout the reaction. Anisotropy changes were examined up to 150 ms. In order to determine the temperature dependent association rate constants, the temperature of the flow-cell and solution reservoir were maintained using a temperature controlled circulating water bath. IRP1 protein binding induced an increase in ^FI^IRE-RNA anisotropy. After rapid mixing of 0.1 µM (0.05 µM final) FRT or ACO2 ^FI^IRE-RNA with 1 µM (final) of IRP1 protein, the time course of the anisotropy change was recorded by computer data acquisition. Samples were degassed prior to loading into the syringes. All measurements were performed in titration buffer containing 40 mM HEPES/H^+^, pH 7.2, 100 mM KCl. The stopped-flow traces shown under “Results” are the average of 5–7 individual shots to improve the signal-to-noise ratio. Each averaged set of stopped-flow anisotropy data was then fitted to nonlinear analytical equations using GlobalWorks^TM^ analysis software (OLIS).

In order to measure the effect of Mn^2+^ on the temperature dependent FRT and ACO2 IRE-RNA binding with IRP1, 50 µM Mn^2+^ was added to both IRE-RNA and protein solutions, at the same concentration, and the solutions were incubated separately for 15 min before adding to titration buffer containing the same metal ion concentration as the IRE-RNA and protein solutions (50 µM Mn^2+^). Experiments were performed as described above. Kinetic data were evaluated by fitting to the single-exponential functions as described elsewhere^29, 47–49^ and further analyzed as described below.

### Curve Fitting and Stopped-flow Kinetic Data Analyses

Temperature dependent stopped-flow traces representing binding of FRT IRE-RNA or ACO2 IRE-RNA with IRP1 protein were analyzed using a curve-fitting program, Global analysis software as described previously^47, 50, 51^. Data were fit to the single-exponential functions. Fitted curves correspond to the following single-exponential equation,

4 $${{\rm{r}}}_{({\rm{t}})}-{{\rm{r}}}_{{\rm{f}}}={{\rm{Ae}}}_{{\rm{obs}}}^{-k}\cdot {\rm{t}}$$

where r_(t)_ is the observed anisotropy at any time, t, and r_f_ is the final value of anisotropy, and A is the amplitude. *k*_obs_ is the observed first order rate constant. The reaction was consistent with a single-exponential process. An assessment of each fit was made from the residuals, which measure the difference between experimental data and the calculated fit. The program KaleidaGraph version 2.1.3 (Synergy Abelbeck Software) was used for least-squares fitting of data with linear equations and determination of standard errors for parameters obtained from the fits.

To determine the activation energy of the complexes, FRT or ACO2 IRE-RNA with IRP1 protein in the absence and presence of Mn^2+^, the fitted rate constants were used to construct Arrhenius plots according to the equation

5 $$\mathrm{ln}\,k=(-{E}_{a}/R{\rm{T}})+\,\mathrm{ln}\,A$$

where *E*_a_ is the activation energy, *k* is the rate constant, *R* is the universal gas constant, T is the absolute temperature, and *A* is the Arrhenius pre-exponential factor. The activation energies were calculated using the slopes of the fitted linear plot of ln *k versus* 1/T (kelvin).
